# Calcium functional imaging with high-resolution CT in the inner ear

**DOI:** 10.1038/s41598-021-94857-3

**Published:** 2021-07-27

**Authors:** Hisaya Tanioka, Sayaka Tanioka

**Affiliations:** Tanioka Clinic, Tanioka Bldg. 3F, 6-24-2 Honkomagome, Bunkyo-Ku, Tokyo, 113-0021 Japan

**Keywords:** Biophysics, Computational biology and bioinformatics, Neuroscience, Physiology, Anatomy, Neurology

## Abstract

Although the otolith and otolith organs correlate with vertigo and instability, there is no method to investigate them without harmful procedures. We will create the technique for 3D microanatomical images of them, and investigate the in vivo internal state and metabolisms. The otolith and otolith organs images were reconstructed from a texture synthesis algorithm under the skull volume rendering algorithm using a cutting-plane method. The utricular macula was elongated pea-shaped. The saccular macula was almost bud-shaped. The changes in the amount of CaCO_3_ in the maculae and the endolymphatic sac showed various morphologies, reflecting the balance status of each subject. Both shapes and volumes were not always constant depending on time. In Meniere’s disease (MD), the saccular macula was larger and the utricular macula was smaller. In benign paroxysmal positional vertigo (BPPV), the otolith increased in the utricular macula but did not change much in the saccular macula. The saccule, utricle, and endolymphatic sac were not constantly shaped according to their conditions. These created 3D microanatomical images can allow detailed observations of changes in physiological and biological information. This imaging technique will contribute to our understanding of pathology and calcium metabolism in the in vivo vestibulum.

## Introduction

Movements and balance are controlled by sensorimotor systems as sensory peripheral organs such as vision, tactile, and the vestibular end-organs. Keeping a balance is maintained by the brain’s output from a complex set of sensorimotor control systems inputs, which are visual, muscles, joints, and the vestibular end-organs. The vestibular end-organs play in motion, equilibrium, and spatial recognition. The sensory organ of the vestibular systems consists of the saccular macula, the utricular macula, and three cristae of the superior, lateral, and posterior semicircular canals. The semicircular canal systems identify the circulation movement. The otolith organs as the saccular and utricular maculae detect linear accelerations and decelerations. The utricular signals affect eye movements, and the saccular signals usually go to muscles to maintain our posture^1–4^. Therefore, it can be imagined that these otolith organs may be inconstant morphological shapes in the in vivo inner ear. Little is known about in vivo morphological conditions and their metabolisms, so our knowledge of them is still unclear. If we can know in vivo conditions of the otolith and the otolith organs, we can reveal several unknown functions and metabolisms. The solution to these problems is to establish the in vivo imaging computer technique for thin otolithic membranes from their imaging data set. A texture synthesis algorithm is useful for reconstructing the otolith and the otolith organs. Because texture synthesis is an important technology for graphics and animation and fits for imaging these thin anatomical structures^5^.

We propose the new technique based upon the multi-resolution neighborhood matching technique but the powerful method is effective at synthesizing a wide range of textures. When combined with the volume rendering algorithms^6,7^, these algorithms will create a new algorithm for reconstructing the in vivo otolith and the otolith organs. This imaging technique allows us to simultaneously visualize in the 3D bony labyrinth of the inner ear and the tissue consisting of calcium carbonate (CaCO_3_) within it. This means that we can visualize the morphology of the otolith and otolith organs in the bony labyrinth under normal conditions and their morphological changes under unstable woozy.

The first purpose of this study is to create a new algorithm for reconstructing the otolith and the otolith organs from low-dose temporal bone high-resolution (HR) CT data, analyze the accuracy of the created 3D microanatomical images of the otolith and otolith organs compared to the previous histology books and papers^1–4,8–11^, and visualize the normal state of the otolith and otolith organs. Second, we will investigate the differences in the morphology of the otolith and otolith organs under stable and unstable conditions: benign paroxysmal positional vertigo (BPPV) and Meniere’s disease (MD), and the changes caused by calcium carbonate (CaCO_3_) metabolism.

## Results

We were able to ensure intuitively manipulate any otolith within the inner ear in real-time. The resulting voxel size was 0.18 × 0.18 × 0.50 mm. We used a trapezoidal opacity curve with a morphological texture synthesis algorithm.

### Modeling algorithms and imaging parameters

**Figure 1:**
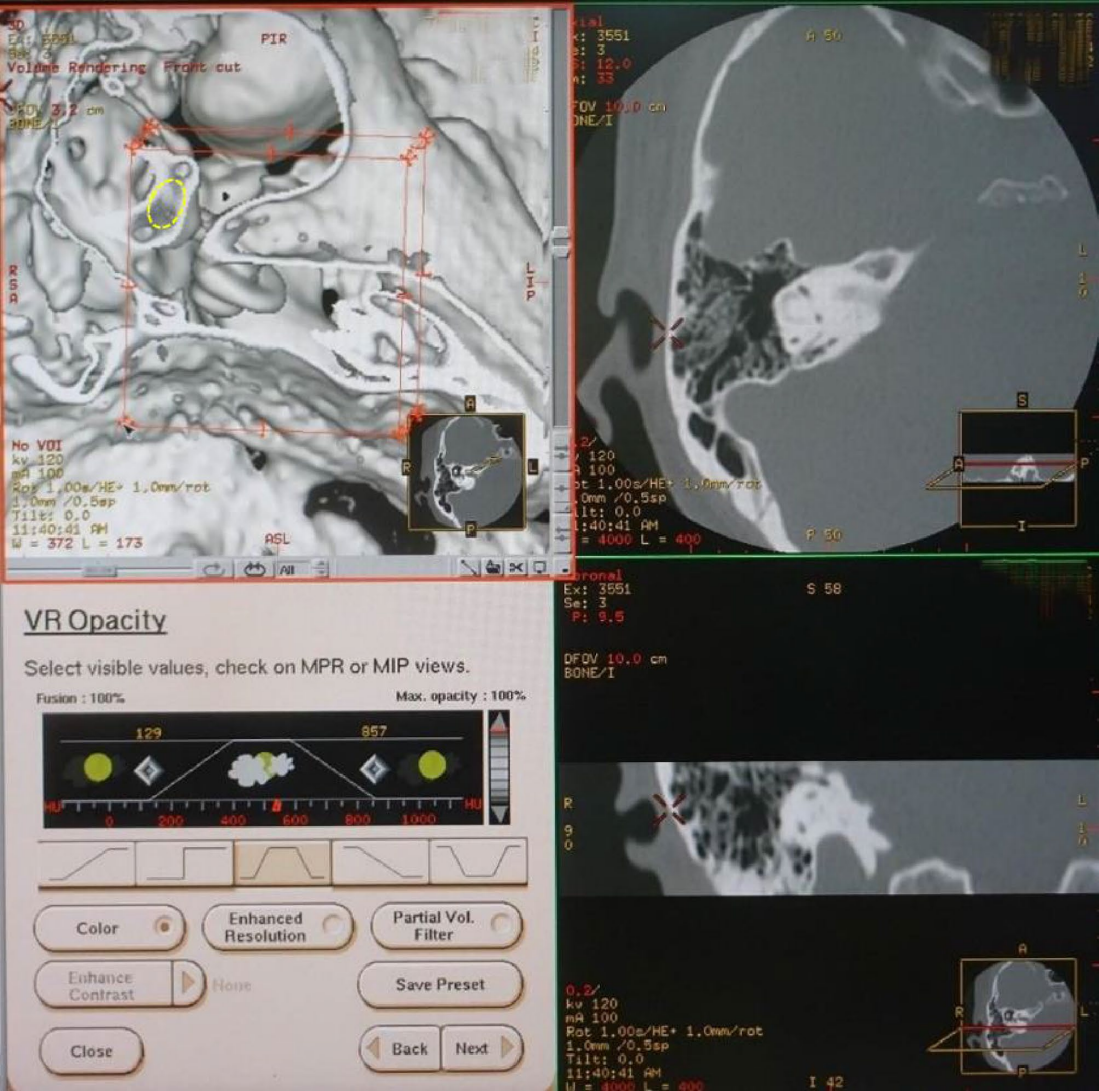
The cutting-plane image on the console processing image for the utricular macula in a 47-year-old female. This image is created by a morphological texture synthesis algorithm. In the upper left image, the normal utricular macula (yellow dotted oval) can be seen inside the bony vestibule.

The specific steps for creating the image were as follows. We created a 3D image of the temporal bone using a 3D skull volume rendering algorithm. It was constructed with pixels instead of voxels. Next, in using a trapezoidal opacity curve, we determined the thresholds for three parts from Table 1^12–16^: 140–400 HU, 400–600 HU, and 600–850 HU. The upward curve portion of the threshold was set to 140 HU to 400 HU. The plateau portion was 400 HU to 600 HU with 100% opacity. The downward curve was set to 600 HU to 850 HU. In the curve section, the opacity value moved according to the threshold. The brightness value was 100%. From the images of the bony labyrinth created by this procedure, the target otolith organ was visualized using the front-cut method. A specific procedure image was presented in Fig. 1.

**Table 1:** CT values of calcium compounds in the human body^12–16^.

Calcium compound	CT value (Hounsfield unit)	
	Range	Mean ± SD
CaCO_3_ (Calcium carbonate)^12^	140–250	189 ± 38.4
Ca_10_(PO_4_)_6_(O)_2_ (Hydroxyapatite)^13,14^	2289–2433	2361 ± 36
CaC_2_O_4_ (Calcium oxalate)^14^	1810–1914	1862 ± 26
CaH[PO_4_]·2H_2_O (Brushite)^14^	2087–2223	2155 ± 34
CaO (Calcium oxide)^15^	2200–3100	2650 ± 83
Ca(OH)_2_ (Calcium hydroxide)^15^	1500–2000	1803 ± 146
NH4MgPO4·6H2O (Struvite)^14^	987–1187	1248 ± 118
Skull^16^	400–850

### Normal morphological imaging of the otolith organs

**Figure 2:**
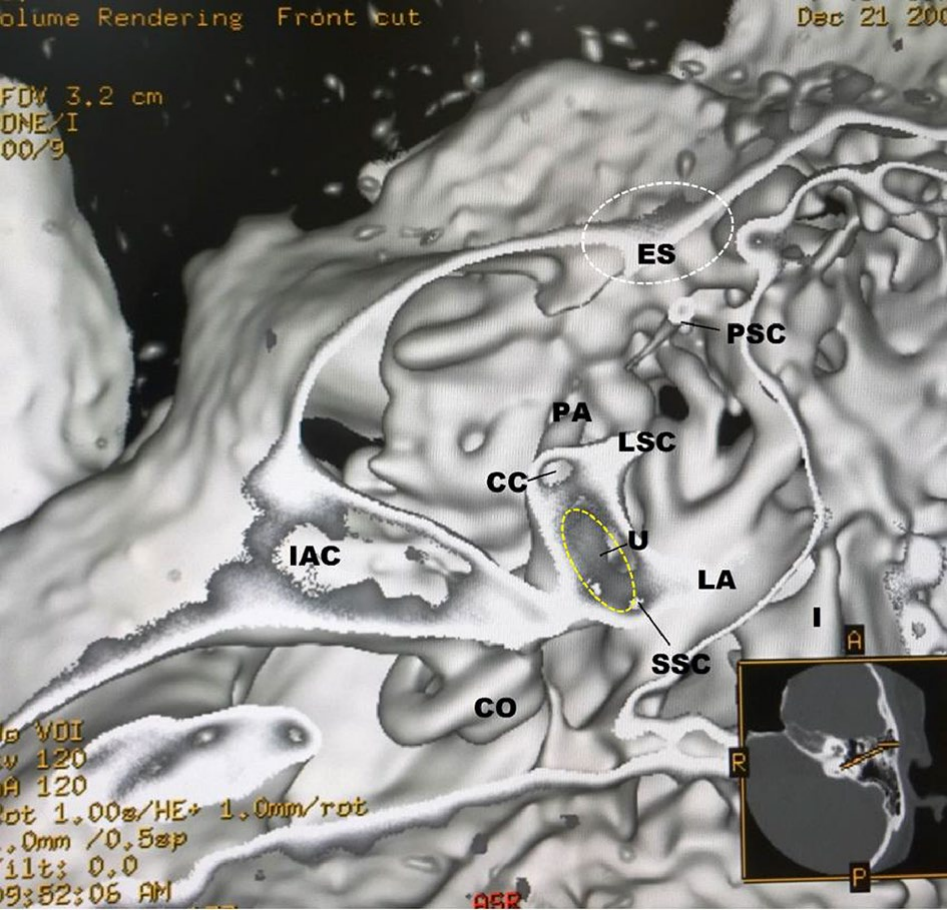
A normal case in a 48-year-old female. From the superior view of the utricular macula, the portion of the utricular macula (yellow dotted oval) lies approximately in the plane of the lateral ampulla and shows a gray peanut-shaped oval. The width and length measured in the 3D image were 1.8 and 2.6 mm, respectively. The endolymphatic sac (white dotted oval) shows a gray triangle. *U* utricular macula (yellow dotted oval), *LA* lateral ampulla, *PA* posterior ampulla, *PSC* posterior semicircular canal, *LSC* lateral semicircular canal, *SSC* superior semicircular canal, *CC* common crus, *ES* endolymphatic sac (white dotted oval), *CO* cochlea, *I* incus, *IAC* internal auditory canal.

**Figure 3:**
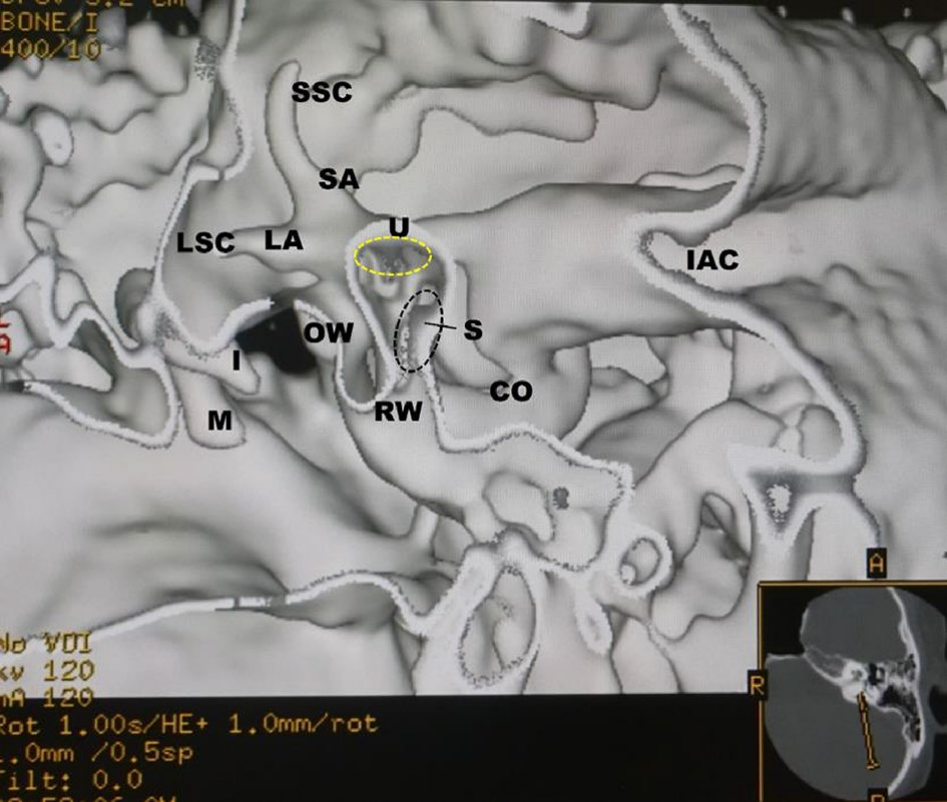
The coronal view from the same subject as in Fig. 2. The utricular (yellow dotted oval) and saccular (black dotted oval) maculae show gray triangles with an irregular contour. *U* = utricular macula (yellow dotted oval), *S* = saccular macula (black dotted oval), *LA* = lateral ampulla, *LSC* lateral semicircular canal, *SA* superior ampulla, *SSC* superior semicircular canal, *CO* cochlea, *I* incus, *M* malleus, *IAC* internal auditory canal, *RW* round window, *OW* oval window.

In the normal cases shown in Figs. 1 and 2, the utricular macula viewed the vestibular axial section from the parietal had a gray peanut-shaped oval occupying the posterosuperior part of the vestibule. The endolymphatic sac had a gray color similar to that of the utricular macula. Figure 3 showed a posterior view of the vestibular coronal section created from the same case as Fig. 2. The saccular macula was bud-shaped and the origin was a trapezoidal shape. The utricular macula was thin and irregularly shaped along the upper vestibular wall. From these cases, the shape and volume of the normal case were different for each subject.

### Clinical imaging of the otolith organs

#### Benign paroxysmal positional vertigo (BPPV) in a 25-year-old female

**Figure 4:**
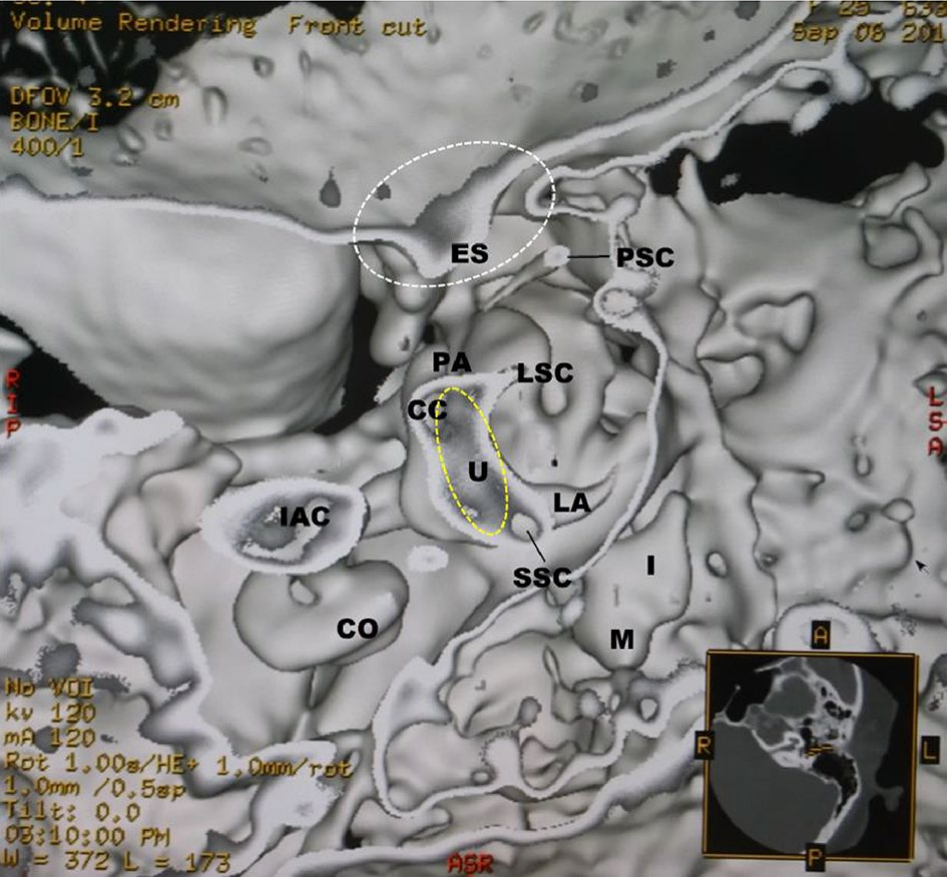
BPPV in a 25-year-old female. In the axial view, the irregularly shaped utricular macula (yellow dotted oval) is seen as a gray tissue extending from near the exit of the superior semicircular canal to the partial exit of the common crus. The endolymphatic sac (white dotted oval) and the cerebellopontine angle cistern show the gray tissue similar to the color of the utricular macula (yellow dotted oval). *U* utricular macula (yellow dotted oval), *PA* posterior ampulla, *PSC* posterior semicircular canal, *PA* posterior ampulla, *LA* lateral ampulla, *LSC* lateral semicircular canal, *SSC* superior semicircular canal, *CC* common crus, *ES* endolymphatic sac (white dotted oval), *CO* cochlea, *IAC* internal auditory canal, *M* malleus, *I* incus.

**Figure 5:**
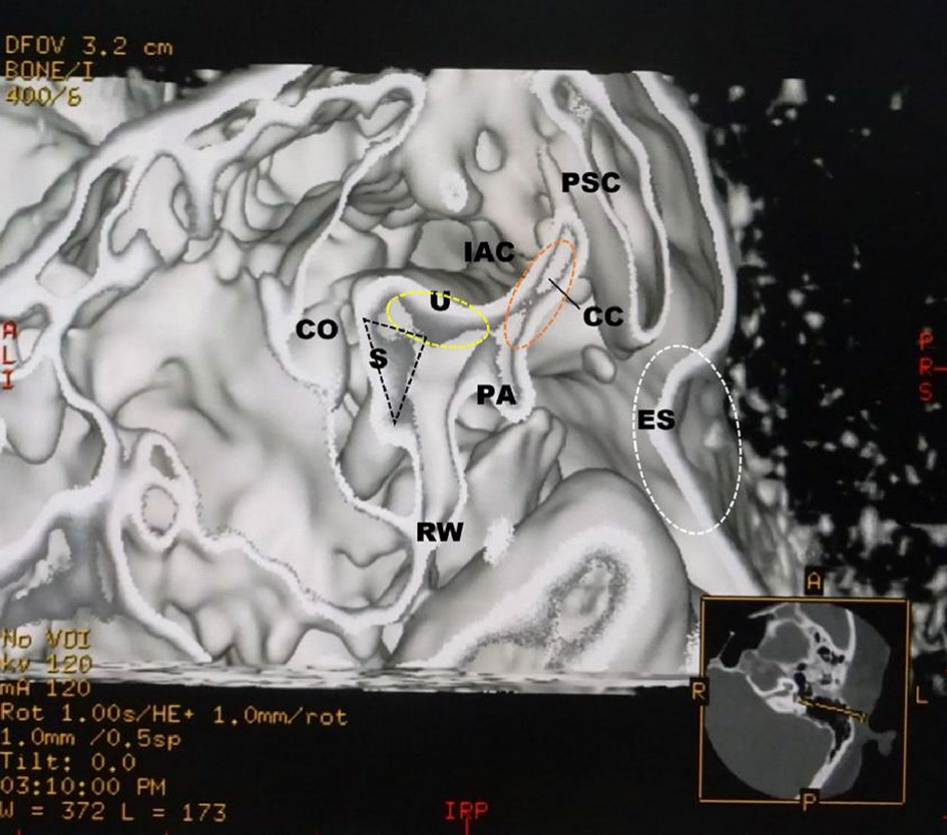
In the parasagittal view from the external auditory canal from the same subject as in Fig. 4, the utricular macula (yellow dotted oval) shows thick and hill-shaped, a small grayish mass is observed from the exit to the body of the common crus (orange dotted oval). The posterior crista ampullaris is gray spindle-shaped. The same gray tissue is detected in the endolymphatic sac (white dotted oval) and peripheral area. *U* utricular macula (yellow dotted oval), *S* saccular macula (black dotted triangle), *PA* posterior ampulla, *PSC* posterior semicircular canal, *CC* common crus (orange dotted oval), *ES* endolymphatic sac (white dotted oval), *RW* round window, *CO* cochlea, *IAC* internal auditory canal.

In the axial vestibular cut view from the parietal of Fig. 4, the irregularly shaped utricular macula was seen as gray tissue extending from near the exit of the superior semicircular canal to the exit of part of the common crus. In the parasagittal vestibular view from the external auditory canal in Fig. 5, the utricular macula became thick and hill-shaped, a small grayish mass was observed from the exit to the body of the common crus. The posterior crista ampullaris was spindle-shaped. The volume and shape of the saccular macula and the crista ampullaris showed almost the same as the normal ones. The same gray tissue was detected in the cerebellopontine angle cistern as in the utricular macula.

#### Meniere’s disease (MD) in a 58-year-old female

**Figure 6:**
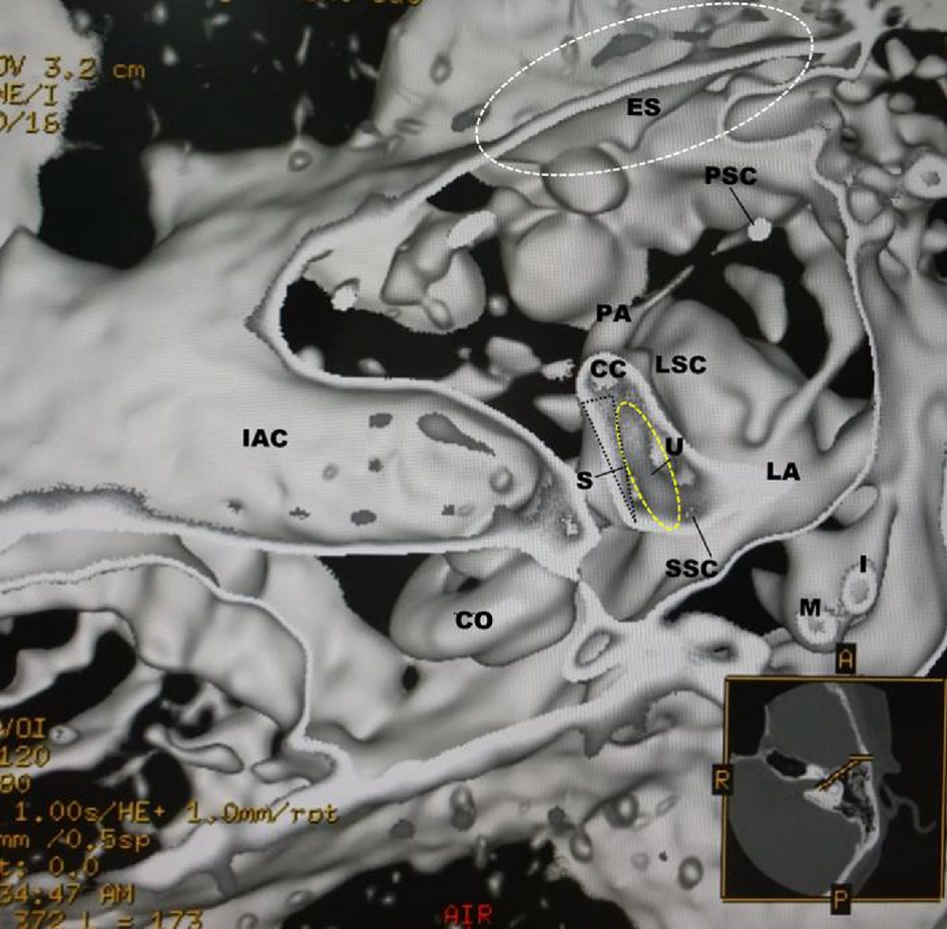
MD in a 58-year-old female. In the axial view, the utricular macula (yellow dotted oval) is in the shape of an elongated pea and connected to the exit of the lateral semicircular canal. The expanded saccular macula (black dotted triangle) is visible from the superior axial view. *U* utricular macula (yellow dotted oval), *S* saccular macula (black dotted triangle), *LA* lateral ampulla, *LSC* lateral semicircular canal, *PA* posterior ampulla, *PSC* posterior semicircular canal, *SSC* superior semicircular canal, *CC* common crus, *ES* endolymphatic sac (white dotted oval), *CO* cochlea, *IAC* internal auditory canal, *M* malleus, *I* incus.

**Figure 7:**
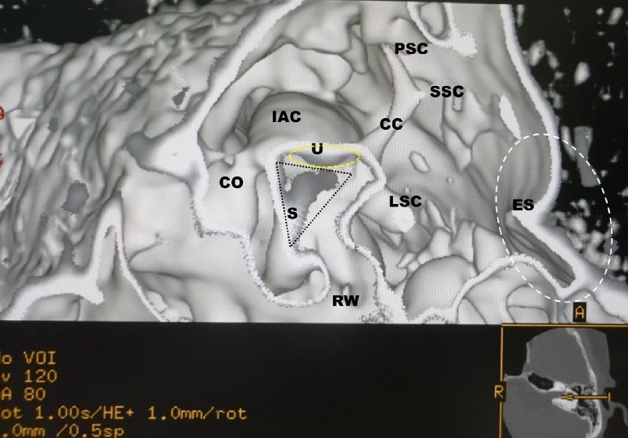
In the parasagittal view from the same subject as in Fig. 6, the saccular macula (black dotted triangle) is enlarged to the extent that it is in contact with the utricular macula (yellow dotted oval). The endolymphatic sac (white dotted oval) is grayish. The same gray-colored tissue is found in the cerebellopontine angle cistern. *U* utricular macula (yellow dotted oval), *S* saccular macula (back dotted triangle), *LSC* lateral semicircular canal, *PSC* posterior semicircular canal, *SSC* superior semicircular canal, *ES* endolymphatic sac (white dotted oval), *CC* common crus, *RW* round window, *IAC* internal auditory canal.

In the axial view of Fig. 6, the utricular macula was elongated and peanut-shaped and connected to the exit of the lateral semicircular canal. In the parasagittal view of Fig. 7, the saccular macula was enlarged to the extent that it was in contact with the utricular macula. The utricular macula showed thinner than normal. The endolymphatic sac was grayish, as was the color of the saccular macula. In the cerebellopontine angle cistern, the same gray-colored tissue as in the maculae and the endolymphatic sac was found more in MD than in other cases.

### The measured results of the otolith organs

The width and height of the saccular otolith membrane were 1.1 ± 0.8 mm and 2.6 ± 0.3 mm, respectively. The width and length of the utricular otolith membrane were 2.2 ± 0.6 mm and 2.7 ± 0.2 mm, respectively. The measured results of ours and those of the previous reports were shown in Table 2. The Welch test showed no statistical significance between the two. The precise p-value was 0.54, 0.61, 0.79, and 0.07, respectively.

**Table 2:** The histological dimensions of the utricle and the saccule^8–11^.

Reporter	Year	Age	Utricle (mm)		Saccule (mm)	
			Width	Length	Width	Length
Corvera et al.^8^	1958		2.1	2.8	1.2	2.2
Beck and Rader^9^	1963		2.0	2.7	1.3	2.4
Rosenhall^10^	1972		2.2	2.8	1.2	2.6
**Takagi and Sando **^11^	1988	5 ma	2.03	2.99	1.36	2.84
		14 years	2.38	3.47	1.64	2.90
		70 years	2.71	3.26	1.38	2.40
		76 years	2.40	2.87	1.32	2.26
Mean			2.28	2.98	1.34	2.51
SD			0.24	0.28	0.15	0.27
Number (n)			7	7	7	7
**This study**						
Mean		50 years	2.2	2.7	1.1	2.6
SD		15	0.6	0.2	0.8	0.3
Number (n)			5	5	5	5

## Discussion

The new algorithm is created using a morphological texture synthesis algorithm combined with a black and white shading algorithm and a trapezoid curve algorithm for the microanatomical imaging of the otolith and otolith organs. In other words, this algorithm is a texture synthesis algorithm under the skull threshold volume rendering algorithm. These images of the in vivo 3D otolith and otolith organs are consistent with the histology books and previous works of histological literature. The measured results of this study and the previous reports were not statistically significantly different. Therefore, the created 3D otolith organs images will be reliable with the in vivo inner ear microanatomy. Although Figs. 1 and 2 are images of a patient of almost the same age, the shape of the utricular macula has not changed much. However, even under normal conditions, the shape and volume of the utricular and saccular maculae vary depending on the subject and age. And, when the subject feels unstable, the size and shape of the utricular macula tend to increase. Our previous study suggests that the vestibular membranous labyrinth may expand with age^7^.

In this study, the endolymphatic sac and its surrounding area show that gray areas, like the color of CaCO_3_, increase when the subject is in an unstable state. Although the functions of the endolymphatic sac are not well understood, these findings seem to suggest that CaCO_3_ excretion by the metabolism of the endolymphatic sac like the previous report^17^. These findings may imply that the inner ear-produced CaCO_3_ may increase with unstable conditions.

For clinical application, we will verify the images of BPPV and MD. In BPPV, the utricular macula is enlarged and the saccular macula is slightly enlarged or almost the same as in normal subjects. On the contrary, in MD, the utricular macula is shrunk and the saccular macula is expanded. These findings indicate that the changes in the status of the utricular and saccular maculae vary depending on the pathology. The endolymphatic sac and its cerebellopontine angle cistern show that the gray areas of CaCO_3_ increase, reflecting the severity of the symptoms.

The images of the otolith and otolith organs created in 3D do not show the same shape or volume across subjects or conditions. Therefore, it would be possible to understand the changes in otolith organs in vivo due to calcium metabolism. In other words, this technique can help us understand the biology of otoliths.

### Radiation dose

In this technique, the Dose Length Product (DLP) is 40–55 mGy/cm, which corresponds to a radiation dose of 0.06–0.08 mSv^18^. This radiation dose is about the same as that of the skull X-P^19^. Therefore, this technique seems to be safe to use in daily practice.

### Limitations

The pixel size resolution of the original data is 180 × 180 µm. On the other hand, the length of otolith is 3 to 19 µm for humans^1^. For this reason, about 10 to 60 otoliths are imaged as one mass. The crista ampullaris has dark cells. In the dark cells of the ampulla, the oval otoliths with a calcium content of about 30% are observed on the utricle side. On the canal side, the round-shaped otoliths with a low calcium content of about 12% are seen^4^. The resulting 3D microanatomical images do not represent different shades that reflect calcium content as shown in Fig. 7. In the current study, the changes in the in vivo otolith organs due to the difference in calcium content cannot be imaged. This cause is probably due to a lack of original accurate data. When creating accurate microanatomical functional images, it is necessary to use higher resolution CT data.

### Artifacts

The otolith organs contain the otolith in the otolithic membrane. The morphological texture synthesis algorithm tends to generate synthetic artifacts in textures that contain unique structural elements with large irregularities such as stone walls^5^. Therefore, this technique needs to understand that these created images have these artifacts around them.

### Threshold problem

The threshold problem in this study is whether the threshold of CT value for CaCO_3_ can be applied to the human body. The elements in the human body are that nearly 99% of the mass consists of just 6 chemical elements of oxygen, carbon, hydrogen, nitrogen, calcium, and phosphorus. Another 5 elements of potassium, sulfur, sodium, chlorine, and magnesium account for most of the last percentage^20^. Therefore, it is reasonable to assume that this defined threshold value for the inner ear represents the calcium compounds presented in Table 1. In addition, considering the composition elements of the endolymph and perilymph fluids, namely, Ca^2+^, Mg^2+^, K^+^, Na^+^, Cl^−^, inorganic phosphorus, CO_2_, and total protein^21^, it will be suggested that the metal compounds in the inner ear are predominantly CaCO_3_ at the defined threshold.

These created 3D images represent rough shapes rather than actual microanatomical images due to artifacts. However, these images will show the CaCO_3_ that makes up the otolith. Therefore, we can know the rough structure and function of the in vivo otolith organs at ease.

In conclusion, we create the new algorithm of volume rendering for the microanatomical images of the otolith and otolith organs. This simplified technique will provide researchers with the tools they need to take basic research to the clinical level. We believe that this technique will allow us to understand the characteristics, metabolism, and functions of the otolith organs in vivo according to their condition.

## Methods

### Participants

This single-center study was obtained by our institutional ethics review board, and all participants gave written informed consent.

Normal temporal bone imaging data from 5 volunteers (2 men and 3 women, mean age 50, range of age: 40–67) who visited for their regular medical checkups were used. These participants had no known affliction of the temporal bone, no hearing and balance problems, and normal findings on both CT exam and physical exam by their medical checkups. Clinical imaging data were obtained from 3 BPPV (1 man; 47-year-old and 2 women; 25 and 65-year-old) and 4 MD (2 male; 58 and 67-year-old and 2 women; 58 and 61-year-old). All participants were normotensive, non-smokers, and free of respiratory, cardiovascular, neurological diseases. They had a follow-up low-dose temporal bone HRCT exam with their annual medical checkups. As for the compared normal histological subjects, the results of the previous histological works were used^1–4,8–11^.

### CT protocol

All examined were performed with a spiral CT scanner (ProSpeed AI; General Electric Systems, Milwaukee, Wis., USA) by using an axial technique with 60–120 kV, 60–120 mA and 60 s scanning time. The section thickness was 0.5 mm. The axial images were reconstructed with a high-resolution bone algorithm in steps of 0.5 mm, a field of view (FOV) of 96 × 96 mm using a 512 × 512 matrix. Radiation doses from CT scans were recorded as displayed on the CT monitor (from the manufacturer’s software; General Electric Medical Systems). The range of dose length product (DLP) was 40 mGy/cm to 55 mGy/cm.

### Imaging algorithms and parameters

#### Morphological texture synthesis algorithm

The otoliths on the otolithic membranes are targeted. The otolithic membranes are embedded in the membranous vestibule. So, the target imaging subjects are the otoliths on the thin membrane composed mainly of CaCO_3_. Hence, the black scale extension exit is most suitable. We need to identify all the regions of the vestibule, and identify a seed pixel of each region using two structure elements B1 (the otolithic membrane) and B2 (the otoliths) to find a given foreground and background configuration, respectively.

That is, we use Hit-Miss Transform as follows.

$$ {\text{HMT}}_{{\text{B}}} \left( {\text{X}} \right) = \left\{ {{\text{x}}\left| {\left( {{\text{B}}_{1} } \right)} \right._{{\text{X}}} \subseteq {\text{X, }}\left( {{\text{B}}_{{2}} } \right)_{{\text{X}}} \subseteq {\text{X}}} \right\}. $$

We need to extract the contours of the otoliths’ aggregate. Therefore, we apply a morphological thickening operation of an extracted contour with a suitable structuring element and the result is stored. The contour segmentation segments the original image with the thin contours. The thickened image retains its original shape. The thickening operation is calculated by converting the origin of the structuring element into each possible pixel position in the image, and at each such position comparing it with the underlying image pixels. In other words, the 3D curvature morphological approach. When the foreground pixel and the background pixel of the structured element completely match the foreground and the background pixels of the image, the image pixel below the origin of the structured element is set as the foreground^5^.

#### Black-and-white shading algorithm

The two colors used for a binary image are black and white. The color used for the object in the image is the foreground color while the rest of the image is the background color.

#### Opacity curve algorithm

A trapezoidal curve opacity algorithm is used to display structures with voxel values within a defined range^6,7^.

#### Threshold values

Thresholding extracts a range of interest by selecting a range region of interest by selecting a range of voxel values that present a specific tissue. The otoliths are made of CaCO_3_. Therefore, only CaCO_3_ is extracted from other calcium compounds in the human body. Table 1 summarizes the values of calcium compounds based on the results of previous literature and published abstracts^12–17^. The CT value range for CaCO_3_ is 140 HU to 250 HU (189.0 ± 38.4 HU)^12^. Hence, the lowest threshold value is 140 HU. When creating the otolith organs with the bony labyrinth, the highest threshold value is the CT value of the bony labyrinth. On the other hand, the minimum skull CT value is about 400 HU, and about 850HU is the average CT value of the skull under 55 years old from the result of the previous literature^17^. Therefore, the maximum threshold CT value is the value of the skull in the range of 400 HU to 850 HU. The trapezoidal curve has three parts: an upward curve, a plateau portion, and a downward curve. In this case, the upward curve portion of the threshold range is set at 140–400 HU from the minimum CaCO_3_ value to the minimum skull CT value. The plateau portion is 400 HU to 600 HU with 100% opacity. 600HU is the average CT value for a 75-year-old skull. This value is considered to be the maximum CT value for skulls covering almost all ages. The downward curve is set to 600 HU to 850 HU. Therefore, it is considered to be the optimal value for skulls of all ages. In the curve section, the opacity value moves according to the threshold.

#### Opacity value

Opacity values are determined to define the relative transparency of each material. Opacity can be turned from 0 to 100%. High opacity values create an appearance like surface rendering. Low opacity value creation can be very useful for seeing things within the lumen^6,7^.

#### Brightness value and cutting-plain method (front cut)

The brightness value is defined as a constant value of 100%. The cutting-plain is a horizontal, vertical, or oblique image of the otolith and otolith organs, as in Fig. 1^22^.

### Postprocessing

After transferring the imaging data to the CT workstation (GE Medical Systems, Milwaukee, Wis., USA), 3D visualization based on interactive direct volume rendering was made using General Electric Advantage Navigator Software; Ver. 2. As a result of this factory preset software of the skull algorithm, both color and opacity values were adjusted interactivity to delineate all structures related to the temporal bone in real-time. Next, as shown in Fig. 1, we created the 3D bony labyrinth under the defined threshold using the trapezoidal opacity curve algorithm that does not display voxel values. The otolithic membranes were created from the bony labyrinth using the cutting plane method (front cut) in the appropriate setting. The display field of view (DFOV) was set to 16–32 × 16–32 mm. The software allowed both distance measurements and volume directly within the 3D scene on the monitor. The workstation color monitor display consisted of 1920 × 1080 pixels, 0.265 mm pixel pitch, and 596.600 × 336.000 mm in size.

This software was almost the same as the open-source ImageJ software (available at https://imagej.nih.gov/ij/download.html) or OsiriX Lite software (available at http://www.osirix-viewer.com).

### Image analysis

Two specialists evaluated the created images by both qualitative and quantitative analyses. One radiologist specialized in head and neck imaging had 30 years of experience, and one researcher had 4 years of experience.

### Qualitative analysis

The qualitative image evaluation was determined by visualization of the following microanatomical structures mentioned in the anatomical books and the previous works of literature^1–4,8–11^. The main evaluation structures were the utricular macula (U), the saccular macula (S), the crista ampullaris (CA), the cochlea (CO), the round window (RW), the oval window (OW), and the endolymphatic sac (ES). Abbreviations in parentheses were shown.

### Quantitative analysis

#### Accuracy of 3D microanatomical image

This study demonstrated whether these images were a significant level of accuracy achievable with this technique. The reconstructed tissue images in normal subjects were measured to ascertain whether these created 3D microanatomical images histologically match. Therefore, the measurements were statistically compared with ours and those of the previous histological literature, as shown in Table 2^8–11^.

### Statistical analysis

Each result was presented as a mean value ± standard deviation.

To compare whether the measured results between this study and the previous histological reports were statistically equal, we carried on the Welch test. A p-value of < 0.05 was regarded as significant. Data were analyzed statistical software available through Microsoft Excel 2016 (Microsoft Corporation Redmond, Washington).

### Guarantor

The scientific guarantors of this publication are HT and ST.

### Statistics and biometry

No complex statistical methods were necessary for this paper.

### Informed consent

All participants gave written informed consent.

### Ethical approval

All procedures performed in studies involving human participants were accorded with the Ethics Committee of Tanioka Clinic, and with the 1964 Helsinki Declaration and its later amendments or comparable ethical standards. Institutional Review Board approval at our clinic was obtained.

### Methodology

Retrospective; experimental, performed at one institution.

## Abbreviations

3D: Three-dimensional
CT: X-ray computed tomography
HRCT: High-resolution X-ray computed tomography
FOV: Field of view
DFOV: Display field of view
HU: Hounsfield units
DLP: Dose length product
VR: Volume rendering
CaCO_3_: Calcium carbonate
BPPV: Benign paroxysmal positional vertigo
MD: Meniere’s disease
